# Social distancing in response to the novel coronavirus (COVID-19) in the United States

**DOI:** 10.1371/journal.pone.0239025

**Published:** 2020-09-11

**Authors:** Nina B. Masters, Shu-Fang Shih, Allen Bukoff, Kaitlyn B. Akel, Lindsay C. Kobayashi, Alison L. Miller, Harapan Harapan, Yihan Lu, Abram L. Wagner

**Affiliations:** 1 Department of Epidemiology, University of Michigan, Ann Arbor, MI, United States of America; 2 Department of Health Management & Policy, University of Michigan, Ann Arbor, MI, United States of America; 3 Independent Consultant, Bloomfield Hills, MI, United States of America; 4 Department of Health Behavior & Health Education, University of Michigan, Ann Arbor, MI, United States of America; 5 Medical Research Unit, School of Medicine, Universitas Syiah Kuala, Banda Aceh, Indonesia; 6 Tropical Disease Centre, School of Medicine, Universitas Syiah Kuala, Banda Aceh, Indonesia; 7 Department of Microbiology, School of Medicine, Universitas Syiah Kuala, Banda Aceh, Indonesia Universitas Syiah Kuala, Banda Aceh, Indonesia; 8 Key Laboratory of Public Health Safety (Ministry of Education), Department of Epidemiology, Fudan University, Shanghai, China; University of South Carolina, UNITED STATES

## Abstract

In order to reduce the spread of SARS-CoV-2, much of the US was placed under social distancing guidelines during March 2020. We characterized risk perceptions and adherence to social distancing recommendations in March 2020 among US adults aged 18+ in an online survey with age and gender quotas to match the general US population (N = 713). We used multivariable logistic and linear regression to estimate associations between age (by generational cohort) and these outcomes. The median perceived risk of infection with COVID-19 within the next month was 32%, and 65% of individuals were practicing more social distancing than before the outbreak. Baby Boomers had lower perceived risk than Millennials (-10.6%, 95% CI: -16.2%, -5.0%), yet were more frequently social distancing (OR = 1.64; 95% CI: 1.05, 2.56). Public health outreach should focus on raising compliance with social distancing recommendations, especially among high risk groups. Efforts to address risk perceptions alone may be inadequate.

## Introduction

Beginning in January 2020, hundreds of millions of people worldwide have been subjected to limitations in their movement in an effort to stem the spread of severe acute respiratory syndrome coronavirus 2 (SARS-CoV-2) and its associated viral disease: COVID-19 [1, 2]. By mid- to late-March 2020, one-fourth of the US population was under a shelter-in-place order [3], and this rapidly increased to include most of the country by April [4].

“Social distancing”, or maintaining a physical distance of at least 2 meters (6 feet) is one way to limit the virus’ transmission, which spreads through respiratory droplets [5, 6]. The World Health Organization (WHO) [7] and the US Centers for Disease Control and Prevention (CDC) [8] both recommend social distancing, as it is one of the only effective tools to reduce the spread of COVID-19 in the absence of a vaccine or effective therapeutics [9]. Widespread social distancing can also provide much-needed time to prepare for the production and allocation of the Personal Protective Equipment (PPE), ventilators, testing kits, and will ‘flatten the curve’ by delaying and reducing the peak number of cases [9].

Recent pleas by public health officials for social distancing and sheltering-in-place during the COVID-19 pandemic have received imperfect public compliance, even when mandated by state or local governments [10]. It is often difficult to encourage an individual to change their mind or adopt new attitudes and behaviors [11]. Anecdotal evidence suggests that political, cultural, and age/generational differences are major contributors to noncompliance [12], but further research is needed to verify, understand, and address these factors. Additionally, risk of severe infection from SARS-CoV-2 is dependent upon age, with older individuals typically experiencing more severe disease and higher mortality [13]. As such, it is important to glean insight from age-specific risk perceptions and social distancing behaviors to help inform recommendations to improve population adherence to social distancing, as well as epidemiological transmission models to predict the course of the pandemic [14].

The COVID-19 pandemic is rapidly evolving and individual behaviors are constantly changing over time, yet it is important to understand how individuals living in the US have perceived the threat of COVID-19 early in the course of the pandemic, and how they have practiced social distancing to determine patterns in contact-reduction. Understanding the features of these behaviors at the beginning of an epidemic provides essential baseline data, as we expect such attitudes and behaviors to change over time as the peak of the outbreak in the U.S. approaches [15–17]. We thus aimed to characterize US adults’ social distancing behaviors in the early phase of the COVID-19 pandemic, and to estimate age-specific differences in risk perceptions and social distancing behaviors by generational cohort.

## Materials and methods

### Study population

In this cross-sectional study, adults over 18 years of age living in the US were sampled from an online survey research firm, Dynata, from March 20^th^ to 22^nd^, 2020. Dynata maintains a sampling frame, into which participants are recruited through social media, websites, and direct email messages [18]. The survey was closed and only open to the Dynata sampling frame. We set age and gender sampling quotas to diversify the sample by age and gender. We report our methods and results in line with the Checklist for Reporting Results of Internet E-Surveys (CHERRIES) [19].

We sought a sample size of 800 such that the margin of error would be 4%, sufficiently precise for estimation with an alpha of 0.05 and a power of 80%, and a proportion of 50% practicing social distancing (a statistically conservative estimate).

### Questionnaire

We developed a brief questionnaire that included questions on COVID-19 risk perceptions, social distancing behaviors, and demographic characteristics. We conducted a pre-test of the survey items with 16 adults ranging in age from early 20s to late 60s. We adjusted the survey items in response to comments from the pre-testers about their comprehension and appropriateness. The questionnaire was in English only. The questionnaire and dataset are available from figshare: https://doi.org/10.6084/m9.figshare.12250034.

### Outcome variables

The study had two main outcomes: perceived risk of COVID-19 and social distancing behavior. Perceived risk of being infected within the next month was assessed on a scale from 0% to 100%, similar to a previous study on H1N1 influenza [20]. This variable was treated as continuous for analysis.

Social distancing was assessed using a categorical measure comparing behaviors within the past week to before the COVID-19 outbreak. What “before the COVID-19 outbreak” meant to the participants was self-determined; no specific date was provided as regional outbreaks within the US have had varying timing. Participants were asked how often they tried to physically distance themselves more than 6 feet from others outside the home. They could respond they had not been within 6 feet of someone, they had been within 6 feet of someone less often, that there had been no change since the COVID-19 outbreak, or that they had been within 6 feet of someone more often. We dichotomized these choices between those practicing social distancing (i.e., not being within 6 feet of someone, or being within 6 feet of someone less often) versus those not (i.e., having no change since COVID-19 outbreak, or being within 6 feet of someone more often).

There were six additional questions which asked about specific social distancing behaviors: going into work/school, having meetings with colleagues/classmates, meeting with friends, going to a club/bar, going to a restaurant, or going outside with a child. Participants were first asked if they typically did these activities, and then, in a similar scale as the primary outcome, whether they had changed their behavior since the start of the COVID-19 outbreak. These variables were analyzed separately and treated as secondary outcomes.

### Independent variables

The primary independent variable in the analysis was age as determined by the individual’s generational birth cohort. The definitions for generational categories are based on age ranges from Pew Research [21]. Due to a limited number of responses among individuals of the “Silent Generation” (individuals ≥75 years old, born 1928–1945), they were included in the same category as Baby Boomers (56–74 years old, born 1946–1964) for analysis. The three other generations were GenX (individuals 40–55 years old, born 1965–1980), Millennials (individuals 24–39 years old, born 1981–1996), and GenZ (individuals 8–23 years old, born 1997–2012, although only individuals ≥18 years were included in the analysis).

Other characteristics were considered control variables. We asked respondents about race/ethnicity in a series of questions similar to the US Census and the 2019 Behavioral Risk Factor Surveillance System (BRFSS) [22]. Due to small sample sizes in some groups, we pooled race into four categories: non-Hispanic White, non-Hispanic Black, Hispanic, and other. Participants also responded to a question on gender identity, formulated using guidelines from the American Association of Public Opinion Researchers [23]. No one selected an “other” gender in this survey. Respondents answered a question about urbanicity derived from the National Health Interview Survey [24]. Finally, participants self-reported their political affiliation (Republican, Democrat, or Independent).

### Statistical analysis

We ran multivariable regression models corresponding to the two main outcomes: perceived risk and social distancing. For social distancing, logistic regression was used, while perceived risk employed a linear regression model after evaluating the outcome distribution and model for homoscedasticity and linearity. The linear regression model outputted β estimates, which can be interpreted as the percentage point difference across categories. Each model included generation as the primary independent variable and also controlled for participant gender, urbanicity, race/ethnicity, family income, and political affiliation. These variables were chosen based on *a priori* considerations and not based on model fit. Models were specified using generalized linear model (GLM) framework in the multivariable regression models. From each model, we also report the marginal means by generational cohort. The marginal means is the mean value averaged across levels of the covariates. We assessed precision of results through 95% confidence intervals (CI). We used SAS version 9.4 (SAS Institute, Cary, NC) for analysis, and R version 3.6.0 (R Foundation for Statistical Computing, Vienna, Austria) for figures. In the figures which show results by age, we eliminated two individuals who reported being 99 years old. We did not weight the individuals to adjust for a non-representative sample.

### Ethical approval

This study protocol was submitted to the University of Michigan Health Sciences & Behavioral Sciences Institutional Review Board (#HUM#00179335). It was deemed exempt as the survey was anonymous and limited to adults, and we obtained a waiver of documentation of informed consent. Participants were given an information sheet, which explained the risks and benefits of the study and that it would take <10 minutes, and they electronically consented prior to starting the questionnaire.

## Results

In total, 1,068 adults started the survey and filled in at least one screening question: 271 (25.4%) did not respond beyond questions on the start screen, and 50 (4.7%) did not consent, leaving 747 participants (70.0%). We excluded 34 individuals (4.6%) who spent less than 3 minutes on the survey, yielding a final sample size of 713.

The demographic characteristics of the study population are shown in Table 1. The study sample was 54.3% female and about one-third (32.5%) of participants lived in a rural area. In terms of age distribution, 34.1% of the study population was in the Baby Boomer (and Silent) Generation, 31.3% GenX, 24.8% Millennial, and only 9.9% GenZ, who were limited to those ≥18 years. About three-fourths of participants were non-Hispanic White (74.5%), with the rest identifying as non-Hispanic Black (7.0%), Hispanic (7.4%), or other (11.1%). By political affiliation, the population was distributed across those identifying as Republicans (31.8%), Democrats (38.5%), and Independents (29.7%).

**Table 1:** Demographics of online survey panel, United States, March 2020.

Demographic Variable		Count	%
Participant's gender	Male	326	45.7%
	Female	387	54.3%
Participant's residence	Rural	227	32.5%
	Urban	471	67.5%
Participant's generation	Baby boomer and silent generation (≥56 years)	242	34.1%
	GenX (40–55 years)	222	31.3%
	Millennial (24–39 years)	176	24.8%
	GenZ (18–23 years)	70	9.9%
Participant's race/ethnicity	Non-Hispanic White	531	74.5%
	Non-Hispanic Black	50	7.0%
	Hispanic	53	7.4%
	Other	79	11.1%
Monthly family income	<$2,000	140	20.2%
	$2,000-$4,999	198	28.5%
	$5,000-$9,999	212	30.5%
	≥$10,000	144	20.7%
Political affiliation	Republican	216	31.8%
	Democrat	262	38.5%
	Independent	202	29.7%
Compliance with social distancing	No	247	35.0%
	Yes	459	65.0%
Perceived risk of infection within next month	*median (IQR)*	32%	11%-51%

### Perceived risk of infection

The median perceived risk of infection within the next month was 32.0% (interquartile range: 10.6%-51.0%). According to the multivariable linear regression analysis, perceived risk of infection was 6.5% higher among those who lived in rural areas than those who lived in urban areas (95% CI: 2.0%, 10.9%). A monotonic relationship was observed between generation and perceived risk, with perceived risk lowest in Baby Boomers (vs. Millennials: -10.6%, (95% CI: -16.2%, -5.0%)), and GenX (vs. Millennials: -7.1%, (95% CI: -12.6%, -1.5%)). Non-Hispanic Black respondents had 11.5% lower perceived risk (95% CI: -20.1%, -2.8%) compared to non-Hispanic White. Across the lower three categories of family income, there were no significant differences in perceived risk, but those making ≥$10,000 a month believed they had 8.5% higher perceived risk compared to those $2,000-$4,999 (95% CI: 2.4%, 14.5%). Additionally, those who identified as Democrats perceived themselves to have a 5.5% higher risk than those identifying as Independents (95% CI: 0.5%, 10.6%), with no significant difference between Democrats and Republicans.

**Fig 1:**
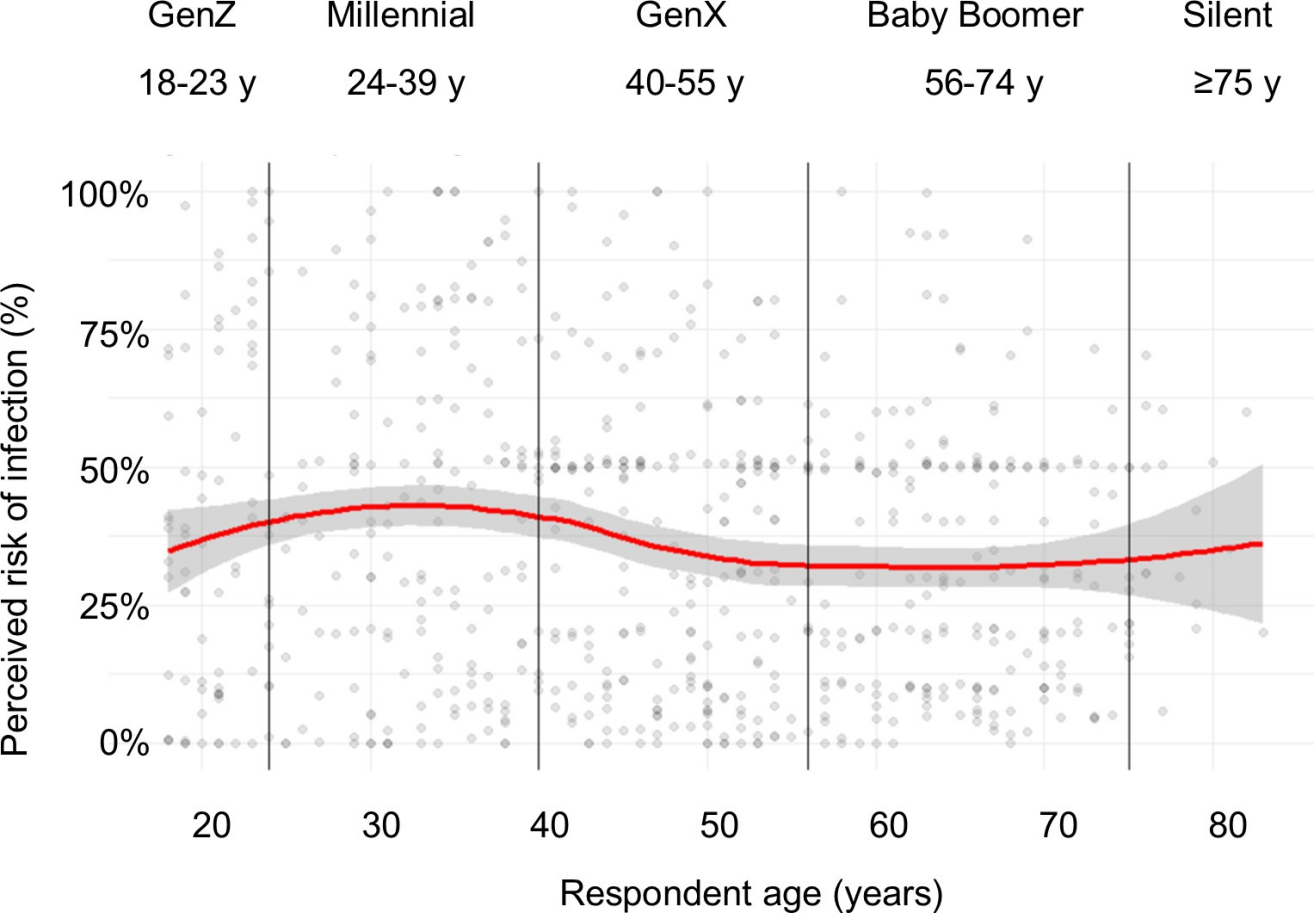
Relationship between age and perceived risk of COVID-19 infection, US, March 2020. Loess line (red) with 95% confidence interval in gray.

Fig 1 shows how risk perceptions varied by age. There was a slight increase between GenZ and Millennials, with decreasing perceptions then in older ages. The marginal mean perceived risk of COVID-19 was 39.8% (95% CI: 33.0%, 46.7%) among GenZ, 40.9% (95% CI: 36.2%, 45.6%) among Millennials, 33.8% (95% CI: 29.0%, 38.6%) among GenX, and 30.3% (95% CI: 25.1%, 35.4%) among Baby Boomers.

### Social distancing

About two-thirds of participants (65.0%) reported practicing more social distancing (staying more than 6 feet away from others) compared to before the COVID-19 outbreak. The odds of social distancing were higher among Baby Boomers (OR = 1.64; 95% CI: 1.05, 2.56) than Millennials, with no difference across other generations. There were no statistically significant differences in social distancing behaviors by gender, urbanicity, race, monthly family income, or political affiliation (Table 2).

**Table 2:** Impact of demographic factors on vaccine hesitancy, novel coronavirus (COVID-19) risk perceptions, social distancing, and COVID-19 vaccine acceptance, online survey panel, US, March 2020.

Demographic Variable		Perceived risk	Social distancing
		β (95% CI)	OR (95% CI)
Participant's gender	Male	ref	ref
	Female	-3.5% (-7.7%, 0.7%)	0.81 (0.58, 1.13)
Participant's residence	Rural	6.5% (2.0%, 10.9%)	0.87 (0.61, 1.23)
	Urban	ref	ref
Participant's generation	Baby boomer and silent generation (≥56 years)	-10.6% (-16.2%, -5.0%)	1.64 (1.05, 2.56)
	GenX (40–55 years)	-7.1% (-12.6%, -1.5%)	1.12 (0.73, 1.73)
	Millennial (24–39 years)	ref	ref
	GenZ (18–23 years)	-1.1% (-9.0%, 6.8%)	0.79 (0.43, 1.44)
Participant's race/ethnicity	Non-Hispanic White	ref	ref
	Non-Hispanic Black	-11.5% (-20.1%, -2.8%)	1.00 (0.52, 1.95)
	Hispanic	-1.0% (-9.2%, 7.1%)	1.04 (0.56, 1.93)
	Other	-1.5% (-9.2%, 6.2%)	1.45 (0.77, 2.73)
Monthly family income	<$2,000	3.6% (-2.4%, 9.6%)	0.67 (0.42, 1.06)
	$2,000-$4,999	ref	ref
	$5,000-$9,999	3.4% (-2.0%, 8.7%)	1.07 (0.70, 1.65)
	≥$10,000	8.5% (2.4%, 14.5%)	1.02 (0.63, 1.66)
Political affiliation	Republican	-0.3% (-5.6%, 5.0%)	0.90 (0.59, 1.37)
	Democrat	5.5% (0.5%, 10.6%)	0.98 (0.65, 1.46)
	Independent	ref	ref

**Fig 2:**
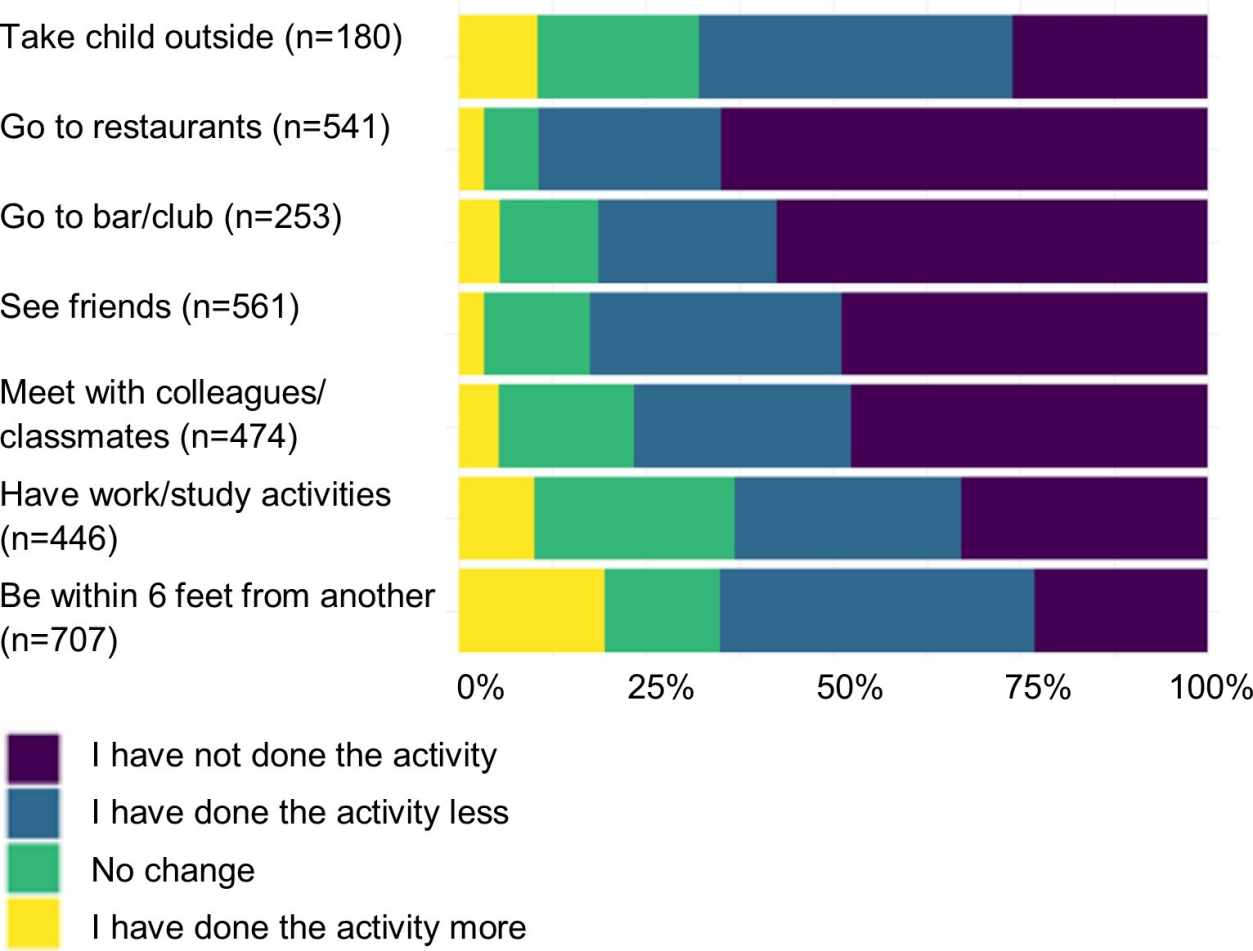
Changes in social distancing behaviors due to the COVID-19 outbreak, US, March 2020.

Large reported changes in usual behavior were observed for those who engaged in specific types of social behaviors regularly prior to COVID-19 (Fig 2). Among those who regularly worked or studied outside of the home (62.0%), 63.0% went out less or not at all after COVID-19 outbreak began. For those who regularly met with colleagues or friends (65.9%), 76.6% met less after COVID-19. Those who would normally regularly meet with friends (78.3%) met significantly less (82.4%) since the COVID-19 outbreak. Among individuals who would go to restaurants (78.3%) or clubs (35.2%), over 80% went to these locations less due to COVID-19. Two-thirds (67.8%) of adults who would regularly go outside with their children (25.2%) were limiting this activity.

**Fig 3:**
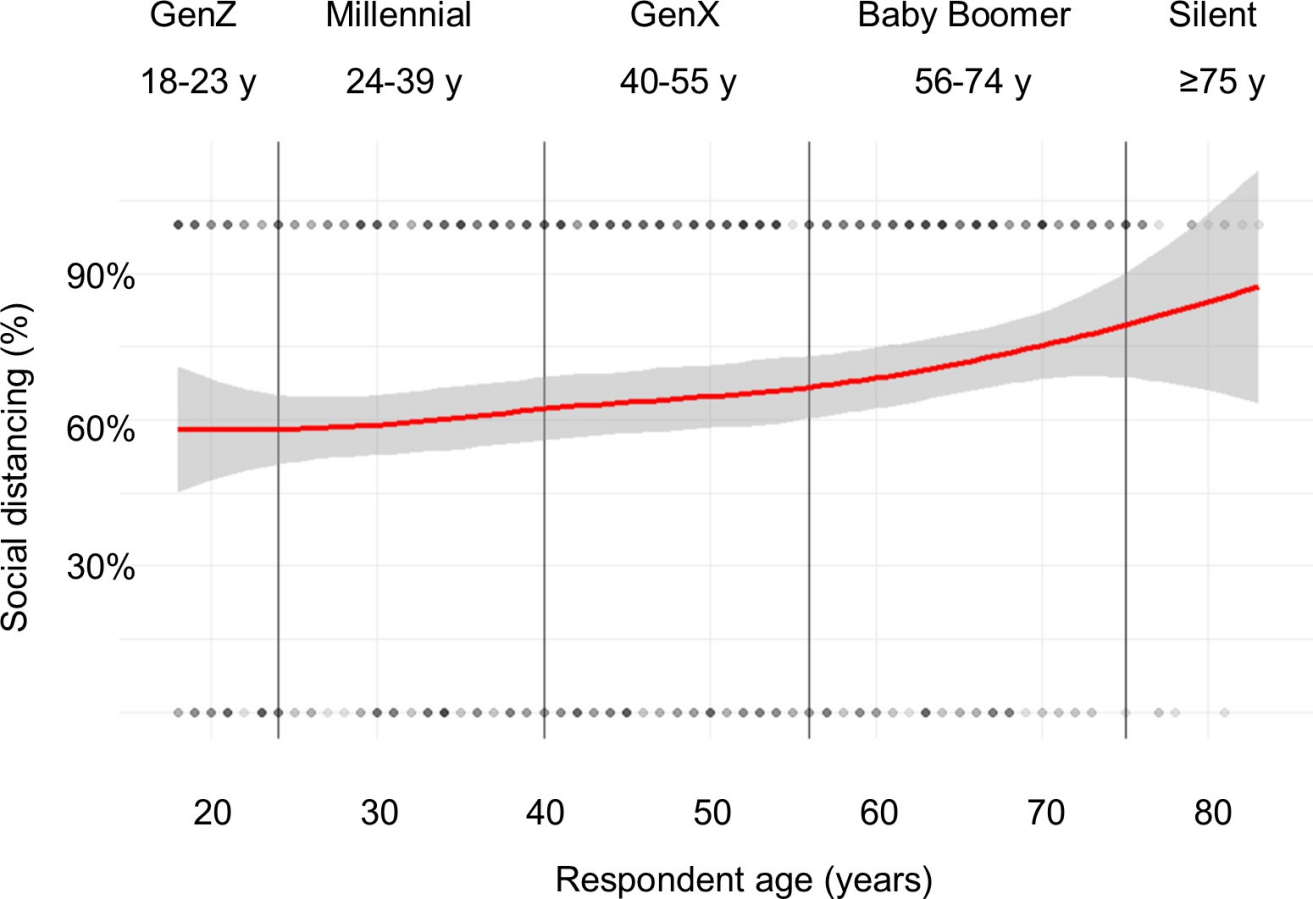
Relationship between age and social distancing behaviors, US, March 2020. Loess line (red) with 95% confidence interval in gray.

Fig 3 shows the relationship between age and social distancing. There was a slight increase in the proportion who were social distancing as age increased. The marginal mean proportion who reported social distancing was 62.2% (95% CI: 53.4%, 70.3%) among GenZ, 62.2% (95% CI: 53.4%, 70.3%) among Millennials, 64.9% (95% CI: 55.9%, 73.0%) among GenX, and 72.9% (95% CI: 64.0%, 80.4%) among Baby Boomers.

## Discussion

This online, cross-sectional study is among the first to examine risk perceptions and social distancing practices in the US at the onset of the COVID-19 outbreak in the United States. Around the time of the survey, large changes occurred in the governmental response to the outbreak: on March 19, the California Governor issued a stay-at-home order [25], and by April, most of the country was under various similar orders [4]. We observed generational cohort differences in perceived risk of infection and social distancing practices. In particular, compared to Millennials, Baby Boomers had lower perceived risk, but greater odds of practicing social distancing behaviors.

Overall, the median perceived risk (32%) was relatively high. Although testing for COVID-19 and serosurveys are not widely available [26], the proportion of the population infected is unlikely to be as high, and the probability of being infected within the next month is likely to be much lower than 32%. Even New York state, one of the hardest hit regions, has found seroprevalence of COVID-19 antibodies to be <15% of the general population [27]. It could be that individuals ignored the time frame (within one month) in the question on perceived risk, or are overestimating their monthly risk. Moreover, it can be difficult for individuals to process risk estimates [28]. One concern from this high perceived risk is that if individuals think their risk of infection is high, but discover that the true risk is actually lower (yet still nonzero), they may grow less compliant with COVID-19 countermeasures overtime. Nonetheless, despite this high perceived risk of COVID-19 infection, our study found that there was still imperfect compliance with public health recommendations for social distancing.

Our study results indicate that a majority of the U.S. adult population was practicing social distancing as of late March 2020. However, a substantial minority (35.0%) were not, which may limit efforts to control the spread of COVID-19, especially given the relatively short time from in which individuals have had to partake in social distancing behaviors so far. By late March, the population may not yet have been experiencing fatigue from extended time indoors and changes to their normal social behavior [29].

The COVID-19 pandemic is forcing everyone to decide–in the face of uncertainty–how to behave, and form attitudes to guide their actions [30, 31]. Uncertainty can have a large impact on perceptions about health risks [32]: when people do not have all the information necessary to make a decision, the effort they put into making the leap to a decision depends on (1) their perception of the gravity of the situation and (2) expectations of the consequences of their actions. People who do not find a health threat to be serious are more likely to make knee-jerk decisions and justify reasoning post-hoc, while those who think it serious are more likely to research and weigh credible sources of information [30, 31, 33]. These factors make effective public health messaging even more important in ensuring that the population broadly considers the risk of COVID-19 to be real and complies with social distancing guidelines.

### Generational and sociodemographic differences

We found that social distancing behaviors varied by generational cohort, with more social distancing practiced in older than younger generations, even though risk perceptions were lower in older groups. Anecdotally, it is thought that younger generations are not taking the virus seriously, with frequent news stories about college students still going on spring break trips [34] and not practicing social distancing [35, 36]. Younger adults also may believe that they are less at risk for severe disease [12]. Although we did find a significant different in social distancing behaviors by generation, it is important to note that overall, more than 60% of adults of all generations were trying to maintain physical distance of 6 feet or more from others, indicating that a majority of the population was engaging in social distancing efforts.

The differences in social distancing by generation could be due to a number of factors. For example, it could be that, even before stay-at-home orders were issued, Baby Boomers responded to initial reports of a high case burden of older adults in long-term care facilities [37]. On the other hand, Millennials had higher perceived risk of infection, but did not practice as many social distancing behaviors, potentially due to other barriers such as jobs, childcare, or housing insecurity, or a lack of understanding of the term ‘social distancing’ [10].

Beyond age, there are other sociodemographic factors which could impact risk perceptions and social distancing behaviors. Overall, it is important to promote social distancing behaviors to protect those who cannot easily engage in them (e.g., essential workers and those of lower socioeconomic status with less ability to decide to stay at home), and consider factors which may make social distancing difficult, such as housing and food insecurity, child care, job security, and health benefits.

Our study observed some differences in perceived risk by race and ethnicity. Early reports, at least in urban areas of the US, have indicated a much higher infection and fatality rate for non-Hispanic Blacks than for non-Hispanic Whites [38]. These differences may be related to income, as those with lower-income jobs are less able to abstain from using public transportation, to be permitted to work remotely, or live in environments that permit social distancing [39]. Our study did find those in the highest income category (>$10,000) to have higher perceived risk. Though emerging data appears to indicate that risk of disease may be higher among non-Hispanic Blacks, their perceived risk in this study was lower. However, it is difficult to interpret the differences in perceived risk, given that they are quite high in general, and so non-Hispanic Blacks’ lower perceived risk may be more realistic. Nonetheless, it is important to consider that minority groups may have less trust in governmental institutions as a result of longstanding structural racism [40, 41]. Accordingly, governmental health promotions should focus on addressing higher risk of severe infection and morality in historically marginalized communities [42, 43].

We did not observe differences by political affiliation. Our finding that individuals of all political parties adhered to social distancing behaviors, despite variable reporting on the severity of COVID-19 and the need for such measures by elected officials, is an important and heartening indication of behavioral compliance. Current political differences and variable messaging between conservatives and liberals in the US appear to contribute to uncertainty and create differences in both fear of COVID-19 and behavioral compliance [10, 12, 44]. In response to stay-at-home orders, there have been political demonstrations, but it is notable that these are, at the current time, poorly attended [45].

### Strengths and limitations

Although the use of an internet-based sample allowed us to rapidly collect responses in a short time period and avoid in-person interactions, such samples may only represent those who have access to the internet, and thus our findings may not be generalizable outside of internet users. As of 2019 in the US, 90% of individuals use the internet, but this proportion varies by age, with only about 73% of those ≥65 years using the internet [46]. Participants who self-selected into the sample may differ from those who did not, even among internet users, which could result in selection bias that is difficult to quantify or speculate about in the absence of measured attributes of non-survey responders. We excluded individuals who quickly completed the survey, but there may be others who completed the survey without much thought. We did not generate a nationally representative sample, and our sample includes more individuals who are non-Hispanic White (74.5%), than the general population (60.4%) [47]. We acknowledge that quota sampling could lead to additional biases, for instance, individuals changing their age or gender to be able to complete the survey [48].

## Conclusions

Overall, we found that perceived risk of infection from COVID-19 in the short term was high– 32%—and a majority of surveyed respondents were adhering to social distancing recommendations, regardless of age, race/ethnicity, and political affiliation. However, we did find some generational differences in behavioral responses to the COVID-19 outbreak in the US, with older adults being more likely to socially distance themselves, but having lower perceived risk of infection. Public health outreach and future epidemiological research on COVID-19 should focus on raising compliance with social distancing recommendations among groups with relatively low adherence to recommendations.

## References

[1] Harapan H, Itoh N, Yufika A, Winardi W, Keam S, Te H, et al Coronavirus disease 2019 (COVID-19): A literature review. J Infect Public Health. 2020;13: 667–673. 10.1016/j.jiph.2020.03.019 32340833 PMC7142680

[2] Lasry A, Kidder D, Hast M, Poovey J, Sunshine G, Winglee K, et al Timing of Community Mitigation and Changes in Reported COVID-19 and Community Mobility—Four US Metropolitan Areas, February 26- April 1, 2020. MMWR Morb Mortal Wkly Rep. 2020;69: 1–7. 32298245 10.15585/mmwr.mm6915e2 PMC7755061

[3] Foster BR, Mundell EJ. U.S. Coronavirus Cases Pass 26,000, With 1 in 4 Americans Under “Shelter-in-Place” Orders. In: U.S.News & Weekly Report [Internet]. 2020 [cited 22 Mar 2020]. Available: https://www.usnews.com/news/health-news/articles/2020-03-22/us-coronavirus-cases-pass-26-000-with-1-in-4-americans-under-shelter-in-place-orders

[4] Secon H, Woodward A. About 95% of Americans have been ordered to stay at home. This map shows which cities and states are under lockdown. In: Business Insider [Internet]. 2020 [cited 20 Apr 2020]. Available: https://www.businessinsider.com/us-map-stay-at-home-orders-lockdowns-2020-3

[5] World Health Organization. Report of the WHO-China Joint Mission on Coronavirus Disease 2019 (COVID-19) [Internet]. 2020 [cited 18 Mar 2020]. Available: https://www.who.int/docs/default-source/coronaviruse/who-china-joint-mission-on-covid-19-final-report.pdf

[6] Centers for Disease Control and Prevention. Coronavirus Disease 2019: Social Distancing, Quarantine, and Isolation [Internet]. 2020 [cited 21 Apr 2020]. Available: https://www.cdc.gov/coronavirus/2019-ncov/prevent-getting-sick/social-distancing.html

[7] World Health Organization. Coronavirus disease (COVID-19) advice for the public [Internet]. 2020 [cited 26 Mar 2020]. Available: https://www.who.int/emergencies/diseases/novel-coronavirus-2019/advice-for-public

[8] Centers for Disease Control and Prevention. How to Protect Yourself [Internet]. 2020 [cited 26 Mar 2020]. Available: https://www.cdc.gov/coronavirus/2019-ncov/prepare/prevention.html

[9] Wilder-Smith A, Freedman DO. Isolation, quarantine, social distancing and community containment: pivotal role for old-style public health measures in the novel coronavirus (2019-nCoV) outbreak. 2020; 10.1093/jtm/taaa020 PMC7107565 32052841

[10] Pinsker J. The People Ignoring Social Distancing. In: The Atlantic [Internet]. 2020 [cited 3 Apr 2020]. Available: https://www.theatlantic.com/family/archive/2020/03/coronavirus-social-distancing-socializing-bars-restaurants/608164/

[11] Albarracin D, Shavitt S. Attitudes and Attitude Change. Annu Rev Psychol. Annual Reviews; 2018;69: 299–327. 10.1146/annurev-psych-122216-011911 PMC12479097 28841390

[12] McKay Coppins. The Social-Distancing Culture War Has Begun. In: The Atlantic [Internet]. 2020 [cited 3 Apr 2020]. Available: https://www.theatlantic.com/politics/archive/2020/03/social-distancing-culture/609019/

[13] Wu Z, McGoogan JM. Characteristics of and Important Lessons From the Coronavirus Disease 2019 (COVID-19) Outbreak in China. JAMA. 2020;323: 1239 10.1001/jama.2020.2648 32091533

[14] Prem K, Liu Y, Russell TW, Kucharski AJ, Eggo RM, Davies N, et al The effect of control strategies to reduce social mixing on outcomes of the COVID-19 epidemic in Wuhan, China: a modelling study. Lancet Public Heal. 2020;2667: 1–10. 10.1016/S2468-2667(20)30073-6 PMC7158905 32220655

[15] Peretti-Watel P, Verger P, Raude J, Constant A, Gautier A, Jestin C, et al Dramatic change in public attitudes towards vaccination during the 2009 influenza A(H1N1) pandemic in France. Eurosurveillance. 2013;18: 1–8. 10.2807/1560-7917.ES2013.18.44.20623 24176658

[16] Ferrante G, Baldissera S, Moghadam PF, Carrozzi G, Trinito MO, Salmaso S. Surveillance of perceptions, knowledge, attitudes and behaviors of the Italian adult population (18–69 years) during the 2009–2010 A/H1N1 influenza pandemic. Eur J Epidemiol. 2011;26: 211–219. 10.1007/s10654-011-9576-3 21476080

[17] Lau JTF, Yang X, Tsui H, Kim JH. Monitoring community responses to the SARS epidemic in Hong Kong: From day 10 to day 62. J Epidemiol Community Health. 2003;57: 864–870. 10.1136/jech.57.11.864 14600111 PMC1732318

[18] Dynata. Panel Book [Internet]. 2020 [cited 29 Apr 2020]. Available: https://www.dynata.com/research-insights/

[19] Eysenbach G. Improving the quality of web surveys: The Checklist for Reporting Results of Internet E-Surveys (CHERRIES). J Med Internet Res. 2004;6: 1–6. 10.2196/jmir.6.3.e34 PMC1550605 15471760

[20] Gidengil CA, Parker AM, Zikmund-Fisher BJ. Trends in risk perceptions and vaccination intentions: a longitudinal study of the first year of the H1N1 pandemic. Am J Public Health. 2012;102: 672–9. 10.2105/AJPH.2011.300407 22397349 PMC3297965

[21] Dimock M. Defining generations: Where Millennials end and Generation Z begins. In: Fact Tank [Internet]. 2019 [cited 26 Mar 2020]. Available: https://www.pewresearch.org/fact-tank/2019/01/17/where-millennials-end-and-generation-z-begins/

[22] Centers for Disease Control and Prevention. 2019 BRFSS Questionnaire [Internet]. 2019 [cited 26 Mar 2020]. Available: https://www.cdc.gov/brfss/questionnaires/pdf-ques/2019-BRFSS-Questionnaire-508.pdf

[23] Wronski L. Why (and how!) to ask survey questions on sexual orientation and gender identity. In: SurveyMonkey [Internet]. 2017 [cited 26 Mar 2020]. Available: https://www.surveymonkey.com/curiosity/ask-survey-questions-sexual-orientation-gender-identity/

[24] Centers for Disease Control and Prevention. Q-Bank [Internet]. 2014 [cited 26 Mar 2020]. Available: https://wwwn.cdc.gov/qbank/QuestionDetailIA.aspx

[25] Office of Governor Gavin Newsom. Governor Gavin Newsom Issues Stay at Home Order [Internet]. 2020 [cited 30 Mar 2020]. Available: https://www.gov.ca.gov/2020/03/19/governor-gavin-newsom-issues-stay-at-home-order/

[26] Heymann DL, Shindo N. COVID-19: what is next for public health? Lancet. 2020;395: 542–545. 10.1016/S0140-6736(20)30374-3 32061313 PMC7138015

[27] New York State. Amid Ongoing COVID-19 Pandemic, Governor Cuomo Announces Phase II Results of Antibody Testing Study Show 14.9% of Population Has COVID-19 Antibodies [Internet]. 2020 [cited 29 Apr 2020]. Available: https://www.governor.ny.gov/news/amid-ongoing-covid-19-pandemic-governor-cuomo-announces-phase-ii-results-antibody-testing-study

[28] LaCour M, Davis T. Vaccine skepticism reflects basic cognitive differences in mortality-related event frequency estimation. Vaccine. 2020;38: 3790–3799. 10.1016/j.vaccine.2020.02.052 32169393

[29] Brooks SK, Webster RK, Smith LE, Woodland L, Wessely S, Greenberg N, et al The psychological impact of quarantine and how to reduce it: rapid review of the evidence. Lancet. Elsevier Ltd; 2020;395: 912–920. 10.1016/S0140-6736(20)30460-8 32112714 PMC7158942

[30] Kahneman D, Slovic P, Tversky A. Judgment under Uncertainty: Heuristics and biases. New York: Cambridge University Press; 1982. 10.1126/science.185.4157.1124 17835457

[31] Gilovich G, Griffin D, Kahneman D. Heuristics and biases: The psychology of intuitive judgment. Cambridge, United Kingdom: Cambridge University Press; 2002.

[32] Ferrer RA, Klein WMP. Risk perceptions and health behavior. Curr Opin Psychol. Elsevier Ltd; 2015;5: 85–89. 10.1016/j.copsyc.2015.03.012 26258160 PMC4525709

[33] Kahneman D. Thinking, Fast and Slow. New York: Farrar, Straus and Giroux; 2011.

[34] Wong CM. Spring Breaker Who Vowed To Party Despite Coronavirus Concerns Has Apologized. In: Huffington Post [Internet]. 2020 [cited 25 Mar 2020]. Available: https://www.huffpost.com/entry/spring-break-brady-sluder-coronavirus-apology_n_5e7a5107c5b63c3b649851f5

[35] Arceneaux M. Young people didn’t social distance because government kept telling them not to worry. In: NBC News [Internet]. 2020 Available: https://www.nbcnews.com/think/opinion/young-people-didn-t-social-distance-because-government-kept-telling-ncna1165281?cid=referral_taboolafeed

[36] Perna MC. Three Ways Millennials And Gen-Z Can Make A Difference Amid Coronavirus. In: Forbes [Internet]. 2020. Available: https://www.forbes.com/sites/markcperna/2020/03/24/three-ways-millennials-and-gen-z-can-make-a-difference-amid-coronavirus/#7755952628af

[37] Kimball A, Hatfield KM, Arons M, James A, Taylor J, Spicer K, et al Asymptomatic and Presymptomatic SARS-CoV-2 Infections in Residents of a Long-Term Care Skilled Nursing Facility—King County, Washington, March 2020. MMWR Morb Mortal Wkly Rep. 2020;69: 377–381. 32240128 10.15585/mmwr.mm6913e1 PMC7119514

[38] Burke D, Fresques H. Early Data Shows African Americans Have Contracted and Died of Coronavirus at an Alarming Rate. In: ProPublica [Internet]. 2020 [cited 14 Apr 2020]. Available: https://www.propublica.org/article/early-data-shows-african-americans-have-contracted-and-died-of-coronavirus-at-an-alarming-rate

[39] Thompson D. The Coronavirus Will Be a Catastrophe for the Poor. In: The Atlantic [Internet]. 2020 [cited 3 Apr 2020]. Available: https://www.theatlantic.com/ideas/archive/2020/03/coronavirus-will-supercharge-american-inequality/608419/

[40] Jamison AM, Quinn SC, Freimuth VS. “You don’t trust a government vaccine”: Narratives of institutional trust and influenza vaccination among African American and white adults. Soc Sci Med. 2019;221: 87–94. 10.1016/j.socscimed.2018.12.020 30576982 PMC6350921

[41] Alsan M, Wanamaker M. Tuskegee and the health of black men. Q J Econ. 2018;133: 407–455. 10.1093/qje/qjx029 30505005 PMC6258045

[42] Sokol R, Fisher E, Hill J. Identifying those whom health promotion hardly reaches: a systematic review. Eval Heal Prof. 2015;38: 518–537. 10.1177/0163278715605883 26405265

[43] Devakumar D, Shannon G, Bhopal SS, Abubakar I. Racism and discrimination in COVID-19 responses. Lancet. 2020;395: 1194 10.1016/S0140-6736(20)30792-3 PMC7146645 32246915

[44] Brownstein R. Red and Blue America Aren’t Experiencing the Same Pandemic. In: The Atlantic [Internet]. 2020 [cited 3 Apr 2020]. Available: https://www.theatlantic.com/politics/archive/2020/03/how-republicans-and-democrats-think-about-coronavirus/608395/

[45] Martina M, Ax J. Scattered protests push back on U.S. coronavirus stay-at-home orders [Internet]. 2020 [cited 21 Apr 2020] pp. 2–5. Available: https://www.reuters.com/article/us-health-coronavirus-usa-protests/scattered-protests-push-back-on-us-coronavirus-stay-at-home-orders-idUSKBN21Y34A

[46] Pew Research Center. Internet/Broadband Fact Sheet [Internet]. 2019 [cited 21 Apr 2020]. Available: https://www.pewresearch.org/internet/fact-sheet/internet-broadband/

[47] United States Census Bureau. QuickFacts: United States [Internet]. 2020 [cited 21 Apr 2020]. Available: https://www.census.gov/quickfacts/fact/table/US/IPE120218

[48] Im EO, Chee W. Quota sampling in internet research: Practical issues. CIN—Comput Informatics Nurs. 2011;29: 381–385. 10.1097/NCN.0b013e3181f9dc45 20975541

